# Physiological Adaptive Strategies of Oil Seed Crop *Ricinus communis* Early Seedlings (Cotyledon vs. True Leaf) Under Salt and Alkali Stresses: From the Growth, Photosynthesis and Chlorophyll Fluorescence

**DOI:** 10.3389/fpls.2018.01939

**Published:** 2019-01-09

**Authors:** Yingnan Wang, Weiguang Jie, Xiaoyuan Peng, Xiaoyu Hua, Xiufeng Yan, Zhiqiang Zhou, Jixiang Lin

**Affiliations:** ^1^Key Laboratory of Saline-alkali Vegetation Ecology Restoration, Ministry of Education, Alkali Soil Natural Environmental Science Center, Northeast Forestry University, Harbin, China; ^2^Department of Food and Environment Engineering, Heilongjiang East University, Harbin, China; ^3^Department of Plant Pathology, North Carolina State University, Raleigh, NC, United States

**Keywords:** *Ricinus communis*, cotyledon, true leaf, salt-alkali stress, chlorophyll fluorescence

## Abstract

*Ricinus communis* is an important energy crop and is considered as one of the most potential plants for salt-alkali soil improvement in Northeast China. Early seedling stage (such as the cotyledon expansion stage) is always a vulnerable stage but plays a vital role in plant establishment, especially under stress conditions. However, little information exists concerning the function of cotyledon and the relationship between cotyledon and true leaf in the adaptation to salt stress and alkali stress of this species. Here, *Ricinus communis* seedlings were treated with varying (40, 80 and 120 mM) salinity (NaCl) and alkalinity (NaHCO_3_), growth, photosynthesis, and chlorophyll fluorescence of cotyledons and true leaves were measured. The results showed that the biomass, photosynthetic parameters, and the q_p_ value of both cotyledons and true leaves decreased with increasing salt-alkali stress, and the decrease in biomass, *g*_s_ and *T*r, of true leaves were much greater than that of cotyledons. Salt-alkali stress only reduced photosynthetic pigments and ΦPSII in cotyledons, but did not affect those in true leaves. Additionally, the Fv/Fm and NPQ between cotyledons and true leaves showed different trends in salinity and alkalinity. The results suggested that alkali stress could cause much more damage to the castor bean seedlings, and different physiological responses and adaptive strategies are found in cotyledons and true leaves under salt-alkali stress. This study will help us develop a better understanding of the adaptation mechanisms of cotyledon and true leaf during early seedling stage of castor bean plant, and also provide new insights into the function of cotyledon in *Ricinus communis* under salt-alkali stress conditions.

## Introduction

Soil salinization and alkalization have been considered as major environmental threats to the agricultural system. They not only inhibit crop growth, but also cause land degradation (Lin et al., 2014; Guo et al., 2015). Arable land acreage all over the world is approximately 1.5 × 10^9^ ha, but 0.34 × 10^9^ ha (23%) of these areas are saline, and 0.56 × 10^9^ ha (37%) are sodic (Yang et al., 2007). For example, in Northeast China, alkalinized land has exceeded 70% of the total area and is still expanding (Wang X. et al., 2011). Previous studies have demonstrated that salt stress and alkali stress are actually two distinct stress types, and the negative effect of alkali stress on plant is much more severe than that of salt stress (Chen et al., 2011; Wang H. et al., 2015). Salt stress generally involves osmotic stress and ion injury. However, alkali stress has the impacts of a high pH level in addition to the same stress features as salt stress, which can inhibit nutrient absorption and result in ionic imbalance in plant cells (Wang H. et al., 2011). To the best of our knowledge, the majority of reports in this field only emphasized the physiological effects of salt stress, with little attention to the alkali stress.

In general, early seedling stage (such as the cotyledon expansion stage) is always vulnerable for plants, especially under stress conditions (Zhou et al., 2014). During this special period, the main function of cotyledons is mobilizing storage materials for the embryonic axis growth, which is defined as the storage-type cotyledon. In addition, some plants have inadequate storage materials and energy in their cotyledons, which is defined as non-storage-type cotyledon. This kind of cotyledon always contains many photosynthetic pigments and is regarded as the photosynthetic organ for the following embryonic axis growth (Ampofo et al., 1976; Schoefs et al., 1994; Schoefs and Franck, 2003; Marshall and Kozlowski, 2010).

So far, the majority of previous studies on the function of cotyledon in seedling development mainly focused on the storage-type cotyledon. Storage substances in this type of cotyledon include a considerable amount of protein, lipids, and polysaccharides (Huang, 1996; Golombek et al., 2001; Hills, 2004). Mobilization of the storage substances from cotyledons can provide amino acid (for *de novo* protein synthesis), fatty acid (for energy), and starch (the most common form of stored carbohydrate for respiration) to the seedlings (Bewley and Black, 1994; Tan-Wilson and Wilson, 2012; Yadav and Bhatla, 2015). However, few studies focused on the photosynthetic activity of plant cotyledon, which is quite important during seedling establishment stage. In an earlier study, Lasley and Garber (1978) reported that cotyledons in *Cucumis sativus* provided 80% of the total net CO_2_ exchange of seedlings but used only 50% of the total photosynthetic area. Some other reports also clarified that cotyledons played an important role in increasing seedling dry weight of *Cynoglossum divaricatum* and *Amaranthus retroflexus* (Zhang et al., 2008). However, the physiological responses and adaptive strategies of plant cotyledons and true leaves under salt-alkali stress during early seedling stage are still poorly understood, especially under alkali stress.

Castor bean plant (*Ricinus communis* L.) belonging to the Euphorbiaceae family is an important oilseed crop all over the world. It is widely cultivated throughout tropical and subtropical regions, such as Brazil, India, China, Thailand, Ethiopia, and Philippines (Ribeiro et al., 2014). This plant has tremendous commercial value because its seeds contain high amount of oil and ranges from 40 to 55% of the seed weight (Scholz and Silva, 2008). The oil is composed of a mixture of triglycerides, which include different types of fatty acids and ricinoleic acids (Armendáriz et al., 2015). Oil extracted from the seed is used to manufacture biodiesel with diverse industrial uses, which can also be used as an alternative fuel to reduce air pollution (Hugoalves et al., 2008; Zhang et al., 2014). In addition, castor bean grows rapidly, has a high leaf area index, and can reach a height of 1–3 m. It is also highly tolerant to salt-alkali soil in the Northeast of China (Zhou et al., 2010). Based on these facts, castor bean is considered to be one of the most potential crops for soil improvement. It is well known that growth and photosynthesis are important for the development and acclimation of early seedling and can directly influence the productivity and fitness of agricultural crops (Schoefs and Franck, 2003; Lin et al., 2016). Castor bean cotyledons play a role in photosynthesis, and the cotyledons of this species do not off from the seedlings for more than 1 month, and is great differed from other dicotyledons, such as soybean(Sebastián et al., 2009) and pea (Hanley et al., 2010). We speculate that castor bean cotyledons have specific functions and different physiological adaptation strategies with the following true leaves, especially under salt-alkali stress.

Therefore, in the present study, we aimed to analyze and compare the physiological responses of cotyledon and true leaf-to-salt stress and alkali stress. Such a scientific problem has never been reported before on the castor bean. To this end, we evaluated the effects of salt-alkali stress on the early seedlings of castor bean (*Ricinus communis*) from the perspective of seedling growth, photosynthesis and chlorophyll fluorescence.

## Materials and Methods

### Plant Material and Stress Conditions

A pot-controlled experiment was carried out in the greenhouse of the Northeast Forestry University in 2017 (45°43′ N, 126°37′ E, Heilongjiang Province, China). The experiment material was Castor bean (*Ricinus communis* L.) var. “Fen Bi 10” (Growing period: 96d; Hundred-grain weight: 35 g; Seed length: 9–14 mm). Seeds were provided by the Shanxi Academy of Agricultural Sciences in China.

The seeds used in the experiment were first germinated (The germination rate was 95%.) in petri dishes (20 cm diameter) containing two layers of filter paper with distilled water. After seeds germinated, seedlings were transplanted into the plastic pots. All the pots were irrigated with a Hoagland nutrient solution once a day, from seedling emergence to 35 days after transplanting. The Hoagland nutrition solution used in this research contained 5.00 mM Ca^2+^, 2.00 mM Mg^2+^, 6.04 mM K^+^, 22.2 μM EDTA-Fe^2+^, 6.72 μM Mn^2+^, 3.16 μM Cu^2+^, 0.765 μM Zn^2+^, 2.10 mM SO_4_^2−^, 1.00 mM H_2_PO_4_^−^, 46.3 μM H_3_BO_3_, 0.556 μM H_2_MoO_4_, and 15.04 mM NO_3_^−^ (Shao et al., 2015). Each pot contained eight seedlings before treatment.

The neutral salt NaCl and alkaline salt NaHCO_3_ were used in the salt and alkali stress groups, respectively. Three concentrations were applied to each stress: 40, 80 and 120 mM. The pH ranges in the salt-alkali stress were 6.20–6.38 and 9.05–9.15, respectively. The pH level in the control group was 6.0.

Thirty five days after transplantation, 28 pots with uniform seedlings were then selected and randomly divided into seven treatment groups, with four pots per set. The control group was watered with the Hoagland nutrition solution only. The stress groups were treated with various concentrations of salt-alkali solution daily, between 5 and 6 p.m. Growth, photosynthesis, and chlorophyll fluorescence change of cotyledons and true leaves were analyzed after 6 days of treatment.

### Growth Measurement

For each plant, cotyledons and true leaves were separated. The fresh weight (FW) of each plant was determined. Cotyledons and true leaves were oven-dried at 105°C for 15 min and then dried at 65°C to a constant weight. The dry weights (DW) were then determined. Water content (WC) was calculated using the following formula: (FW-DW)/DW.

### Gas Exchange Measurement

Cotyledons and the first pair of true leaves from four randomly selected seedlings per pot were chosen to measure net photosynthetic rates (*P_N_*), stomatal conductance (*g_s_*), and the transpiration rate (*T*r) using a portable open flow LI-6400XT gas-exchange system (LI-COR Biosciences, Lincoln, NE, United States) with a Li-6400-02B red/blue LED chamber at 09:00-11:00 before harvest. The PAR was 1000 μmol m^−2^ s^−1^ [the ratio between intensities of red (665 nm) and blue (447 nm) light was 9 to 1] and the ambient CO_2_ concentration, air temperature, and air humidity were approximately 420 μmol mol^−1^, 25°C, and 50%, respectively.

### Chlorophyll Fluorescence

Chlorophyll fluorescence imaging of the cotyledons and true leaves in each treatment was measured with the pulse amplitude modulation chlorophyll fluorescence imaging system (Open FluorCam FC 800-O, Photon System Instruments, Czechia). Twelve cotyledons and twelve true leaves from each treatment were detached, fixed with black paper, and placed under FluorCam CCD camera. The seedlings were dark, adapted for at least 1 h. One pair of LED panels served as a measuring light and an actinic light 1 (red-orange 617 nm in standard version). Other two panels provide actinic light 2 and a saturating pulse (in standard version cool white, typically 6500 K). The intensity of saturating light pulses was 45% (1897.96 μmols^−1^m^−2^), and the intensity of actinic light was 48.95% (1000 μmols^−1^m^−2^).

The resulting fluorescence images were analyzed with the FluorCAM program for calculating fluorescence parameters F_v_/F_m_ (F_v_ = F_m_−F_0_) and the actual quantum yield of photochemical energy conversion in PSII (ΦPSII = [F_m_′−F_t_]/F_m_′). In addition, the Photochemical quenching coefficient (q_P_ = [F_m_′−F_t_]/[F_m_−F_0_]) and non-photochemical quenching coefficient (NPQ = [F_m_−F_m_′]/F_m_′) were calculated according to Roháèek et al. (2008).

### Photosynthetic Pigments

The photosynthetic pigments of the cotyledons and true leaves in each treatment were extracted using 0.1 g of fresh material in 10 mL of 80% acetone at 4°C for 48 h in the dark. The chlorophyll a (Chl a), chlorophyll b (Chl b), and carotenoid (Car) contents were determined with a spectrophotometer (BioMate 3S UV-visible, Thermo Scientific, United States). The concentrations of the pigments were calculated according to the methods described by Arnon (1949).

### Statistical Analysis

The data were analyzed using the statistical software SPSS 13.0 (SPSS Inc., Chicago, IL, United States). The data were subjected to an analysis of variance (one-, two- or three-way ANOVA) with the plant tissue, salinity concentration, salinity type, and their interactions as the sources of variation. *Post hoc* comparisons with Duncan’s multiple range test was used to identify differences between groups with *P* < 0.05 as the significance cut-off.

## Results

### Cotyledon and True Leaf Growth

**FIGURE 1:**
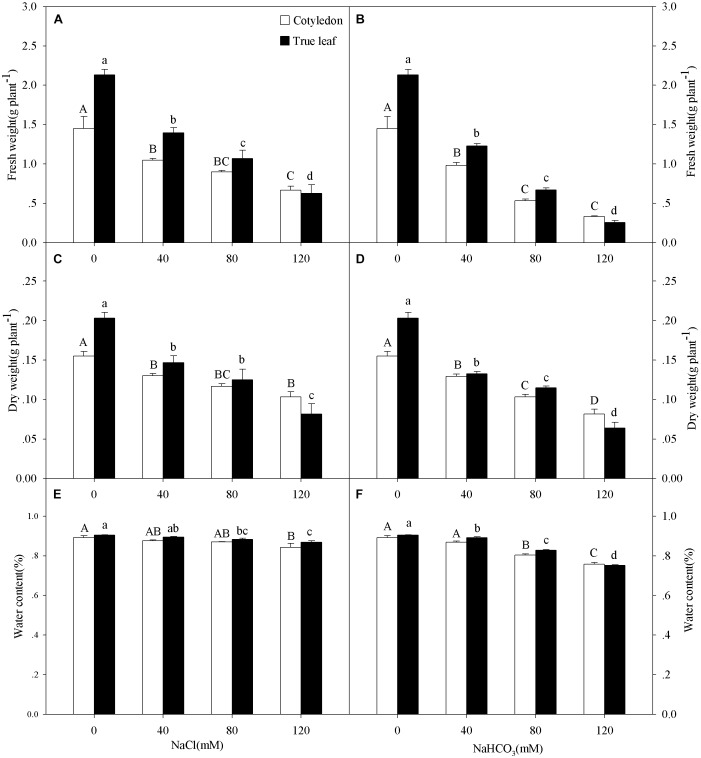
Fresh weight (FW, **A,B**), dry weight (DW, **C,D**), and water content **(E,F)** of *Ricinus communis* cotyledons and true leaves under salt **(A,C,E)** and alkali **(B,D,F)** stresses. Different capital letters indicate a significant difference of cotyledons among the salinity-alkalinity concentrations. Different small letters indicate a significant difference of true leaves among the salinity-alkalinity concentrations. The differences in each parameter were detected by one-way ANOVA at *P* < 0.05 level. Bars represent mean ± SE (*n* = 4).

Three-way ANOVA results showed that the FW, DW, and WC of castor bean seedlings were affected by plant tissue (cotyledon vs. true leaf), salinity concentration, and stress type, but were not affected by interactions of the three factors (Table 1). With increasing salinity concentration, both fresh and DWs of cotyledons and true leaves significantly decreased (*P* < 0.05), and the reduction in true leaves was much higher than that in cotyledons. For example, the fresh and DWs of true leaves decreased by 70.6 and 59.8%, respectively compared to the control group, but only decreased by 54.2 and 33.4% in the cotyledons at the highest concentration (120 mM) of salinity stress (Figures 1A,C). A similar trend of the change in fresh and DWs was also found under alkalinity stress, and the inhibition was markedly higher than that under salinity stress (Figures 1B,D).

**Table 1:** Three-way ANOVA of effects of plant tissue (*PT*), salinity concentration (*SC*), and stress type (*ST*), and their interactions on the growth, photosynthesis, photosynthetic pigments and chlorophyll fluorescence of *Ricinus communis* seedlings.

Dependent variable	Independent variable						
	*PT*	SC	*ST*	*PT* × *SC*	*PT* × *ST*	*SC* × *ST*	*PT* × *SC* × *ST*
FW (g⋅plant^−1^)	51.902^∗∗∗^	231.638^∗∗∗^	32.543^∗∗∗^	17.555^∗∗∗^	0.352^NS^	6.192^∗∗^	0.083^NS^
DW (g⋅plant^−1^)	11.97^∗∗^	133.799^∗∗∗^	7.793^∗∗^	16.001^∗∗∗^	0.057^NS^	1.414^NS^	0.323^NS^
Water content (%)	18.085^∗∗∗^	124.905^∗∗∗^	125.182^∗∗∗^	0.479^NS^	0.361^NS^	41.442^∗∗∗^	1.76^NS^
*P*_N_ (μmol CO_2_ m^−2^s^−1^)	111.723^∗∗∗^	317.868^∗∗∗^	7.469^∗^	4.861^∗∗^	0.259^NS^	0.852^NS^	0.64^NS^
*g*_s_ (mol H_2_O m^−2^ s^−1^)	0.297^NS^	256.812^∗∗∗^	1.945^NS^	19.87^∗∗∗^	8.232^∗∗^	3.785^∗^	4.067^∗^
*T*r (mmol H_2_O m^−2^s^−1^)	0.435^NS^	213.352^∗∗∗^	6.819^∗^	17.775^∗∗∗^	1.826^NS^	0.851^NS^	1.253^NS^
Chl a (mg⋅g^−1^)	620.418^∗∗∗^	7.576^∗∗^	0.456^NS^	0.815^NS^	0.716^NS^	1.427^NS^	1.13^NS^
Chl b (mg⋅g^−1^)	100.134^∗∗∗^	2.178^NS^	0.445^NS^	4.343^∗^	0.624^NS^	3.483^∗^	1.536^NS^
Car (mg⋅g^−1^)	121.576^∗∗∗^	4.473^∗^	9.303^∗∗^	5.822^∗∗^	0.039^NS^	2.904^∗^	1.972^NS^
Fv/Fm (ratio)	217.234^∗∗∗^	18.906^∗∗∗^	0.694^NS^	22.639^∗∗∗^	1.036^NS^	13.121^∗∗∗^	1.922^NS^
ΦPSII (ratio)	180.861^∗∗∗^	13.668^∗∗∗^	2.432^NS^	4.042^∗∗^	0.657^NS^	0.395^NS^	0.738^NS^
q_p_ (ratio)	812.169^∗∗∗^	59.932^∗∗∗^	0.001^NS^	1.575^NS^	15.575^∗∗∗^	3.955^∗∗^	2.627^NS^
NPQ (ratio)	29.794^∗∗∗^	3.001^∗^	2.755^NS^	0.836^NS^	0.104^NS^	0.867^NS^	0.159^NS^

*Date represent F-values at 0.05 level. ^∗^, ^∗∗^, ^∗∗∗^ and NS indicate significance at P < 0.05, P < 0.01, P < 0.001, and P > 0.05, respectively.*

The WC in true leaves decreased significantly under salinity stress, except for the concentration of 40 mM (*P* < 0.05). However, the WC only decreased at the highest salinity concentration (120 mM) in the cotyledons (Figure 1E). With increasing alkalinity concentration, the WC in both cotyledons and true leaves decreased significantly (*P* < 0.05), except for 40 mM alkalinity concentration in cotyledons. In addition, the reductions under alkalinity stress were much greater than that under salinity stress. For example, WC in the cotyledons and true leaves decreased by 13.4 and 15.4%, respectively, at the highest alkalinity (120 mM) compared to the control group, but only decreased by 5.0 and 3.6% at the highest salinity stress concentration (120 mM), respectively (Figures 1E,F).

### Photosynthesis

Three-way ANOVA results showed that net photosynthetic rates (*P_N_*) in castor bean seedlings were affected by plant tissue (*P* < 0.001), salinity concentration (*P* < 0.001), stress type (*P* < 0.05), and the interactions of plant tissue and salinity concentration (*P* < 0.01). The stomatal conductance (*g_s_*) was significantly affected by salinity concentration, and any two or three of these factors. In addition, the transpiration rate (*T*r) in castor bean seedlings was affected by salinity (*P* < 0.001), stress type (*P* < 0.05), and the interactions of plant tissue and salinity (*P* < 0.001; Table 1).

**FIGURE 2:**
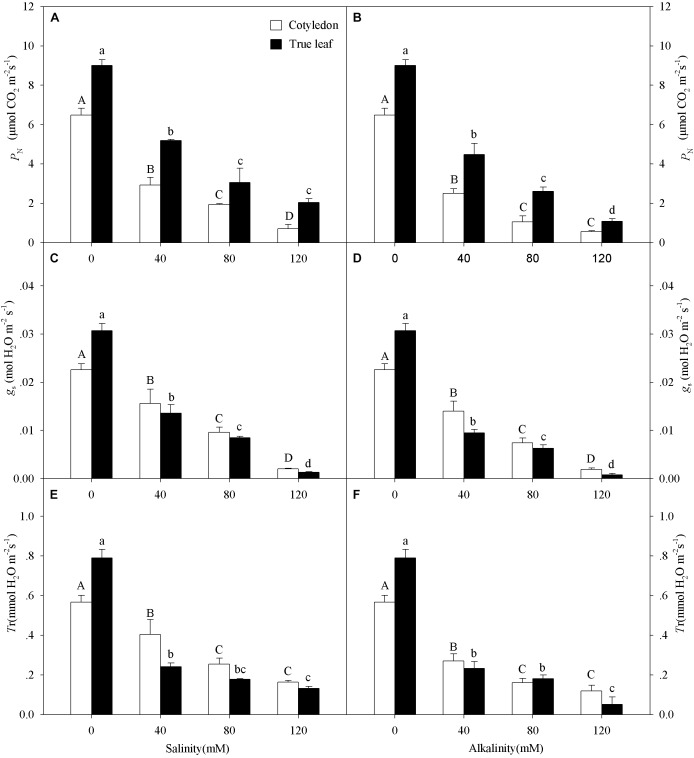
Photosynthetic rate (*P*_N_, **A,B**), stomatal conductance (*g*_s_, **C,D**), and transpiration rate (*T*r, **E,F**) of *Ricinus communis* cotyledons and true leaves under salt **(A,C,E)** and alkali **(B,D,F)** stresses. Different capital letters indicate a significant difference of cotyledons among the salinity-alkalinity concentrations. Different small letters indicate a significant difference of true leaves among the salinity-alkalinity concentrations. The differences in each parameter were detected by one-way ANOVA at *P* < 0.05 level. Bars represent mean ± SE (*n* = 4).

With increasing salinity-alkalinity concentration, the *P_N_*, *g_s_* and *T*r values in both cotyledons and true leaves significantly decreased, and this effect was much more markedly under alkalinity stress (*P* < 0.05, Figure 2). Moreover, the reduction of *P*_N_ in cotyledons was much greater than that in true leaves under salinity-alkalinity stress. For example, the *P*_N_ value decreased by 89.2% in cotyledons and by 77.3% in true leaves at the highest salinity concentration (120 mM), compared to the control group (Figure 2A). However, unlike the *P*_N_ change tendency_,_ the reductions of *g_s_* and *T*r in true leaves were much greater than those in cotyledons under salinity-alkalinity stress.

### Photosynthetic Pigments

Three-way ANOVA results showed that Chl a content in castor bean seedlings was affected by plant tissue (*P* < 0.001) and salinity concentration (*P* < 0.01). The Chl b content was significantly affected by plant tissue (*P* < 0.001), the interactive effect of plant tissue and salinity concentration (*P* < 0.05), and salinity concentration and stress type (*P* < 0.05). Moreover, the Car content in castor bean seedlings was affected by plant tissue, salinity concentration, stress type, the interactive effect of plant tissue and salinity concentration, and the interactive effect of salinity concentration and stress type (Table 1).

**FIGURE 3:**
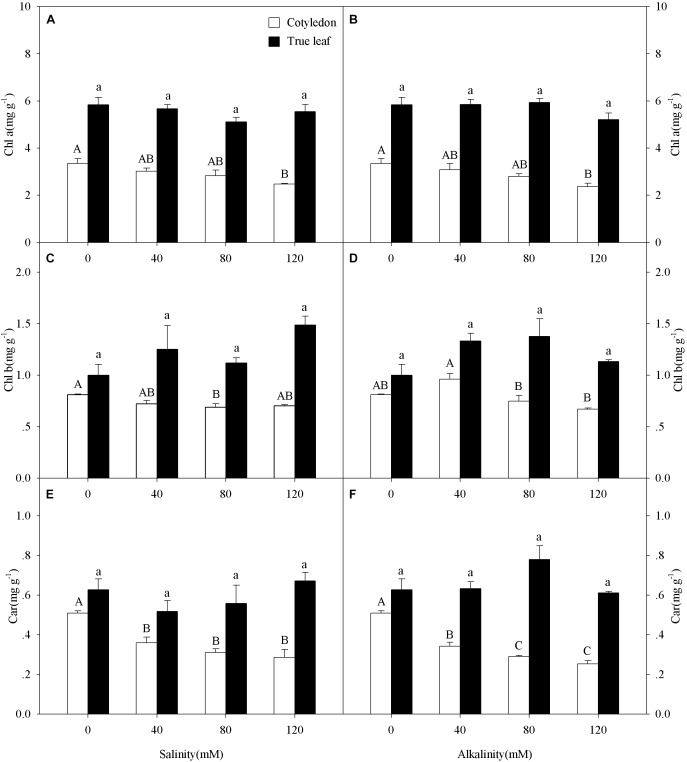
Chlorophyll a (Chl a, **A,B**), chlorophyll b (Chl b, **C,D**), and carotenoid (Car, **E,F**) of *Ricinus communis* cotyledons and true leaves under salt **(A,C,E)** and alkali **(B,D,F)** stresses. Different capital letters indicate a significant difference of cotyledons among the salinity-alkalinity concentrations. Different small letters indicate a significant difference of true leaves among the salinity-alkalinity concentrations. The differences in each parameter were detected by one-way ANOVA at *P* < 0.05 level. Bars represent mean ± SE (*n* = 4).

Chl a content in cotyledons decreased at the highest salinity-alkalinity concentration (120 mM) only (Figures 3A,B), while Chl b content in cotyledons only decreased at 80 mM salinity-alkalinity stress (Figures 3C,D). Car content in cotyledons significantly decreased with increasing salinity-alkalinity stress concentration (*P* < 0.05, Figures 3E,F). However, the Chl a, Chl b, and Car contents in the true leaves remained unchanged with increasing stress concentration (*P* > 0.05). In addition, Chl a, Chl b, and Car contents in true leaves were markedly higher than those in cotyledons under both control and salt-alkali stress conditions.

### Chlorophyll Fluorescence

**FIGURE 4:**
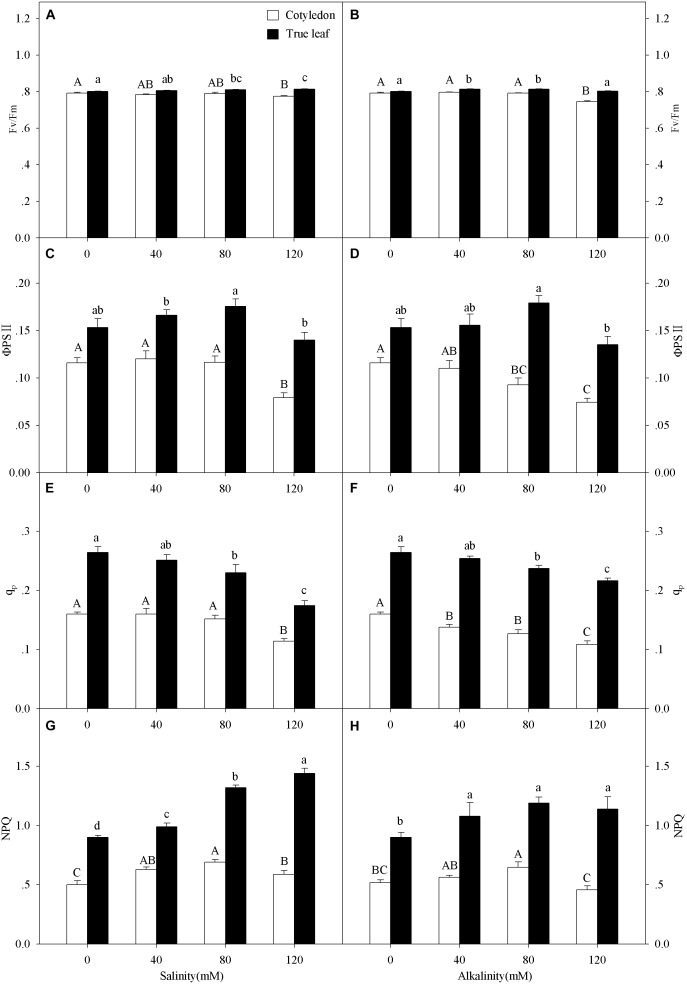
Apparent quantum yield of PSII, (Fv/Fm, **A,B**), actual quantum yield of photochemical energy conversion in PSII (ΦPSII, **C,D**) photochemical quenching coefficient (q_p_, **E,F**) and non-photochemical quenching coefficient (NPQ, **G,H**) of *Ricinus communis* cotyledons, and true leaves under salt **(A,C,E,G)** and alkali **(B,D,F,H)** stresses. Different capital letters indicate a significant difference of cotyledons among the salinity-alkalinity concentrations. Different small letters indicate a significant difference of true leaves among the salinity-alkalinity concentrations. The differences in each parameter were detected by one-way ANOVA at *P* < 0.05 level. Bars represent mean ± SE (*n* = 4).

Three-way ANOVA results showed that Fv/Fm, ΦPSII, q_p_, and NPQ were all significantly affected by plant tissue and salinity concentration, but were not affected by the interactive effects of the three factors (Table 1). With increasing salinity-alkalinity, Fv/Fm in cotyledons decreased at 120 mM stress concentration only, and the reductions were much greater under alkali stress. For example, Fv/Fm in the cotyledons decreased by 2.2% compared to the control group, but decreased by 5.9% at the same concentration of alkali stress. In addition, Fv/Fm in the true leaves showed an uptrend with increasing salinity stress, and only presented significant difference in higher (80, 120 mM) salinity concentrations (*P* < 0.05). Under alkali stress treatments, Fv/Fm in true leaves increased at 40 and 80 mM alkalinities. No significant change was found in 120 mM alkalinity, compared with the control group (*P* > 0.05, Figures 4A,B).

q_p_ values in cotyledons only decreased at the highest (120 mM) salinity stress concentration, but significantly decreased under alkalinity stress (*P* < 0.05). The q_p_ values in true leaves decreased with increasing intensities and reached a significant level when the concentration was above 40 mM at both salt and alkali stresses (*P* < 0.05, Figures 4E,F). ΦPSII in cotyledons only decreased at 120 mM salinity stress, but significantly decreased with increasing alkalinity when the concentration was above 40 mM. Unlike cotyledons, ΦPSII in true leaves remained steady under salt-alkali stress, and the values were much higher than those in cotyledons (*P* < 0.05, Figures 4C,D).

**FIGURE 5:**
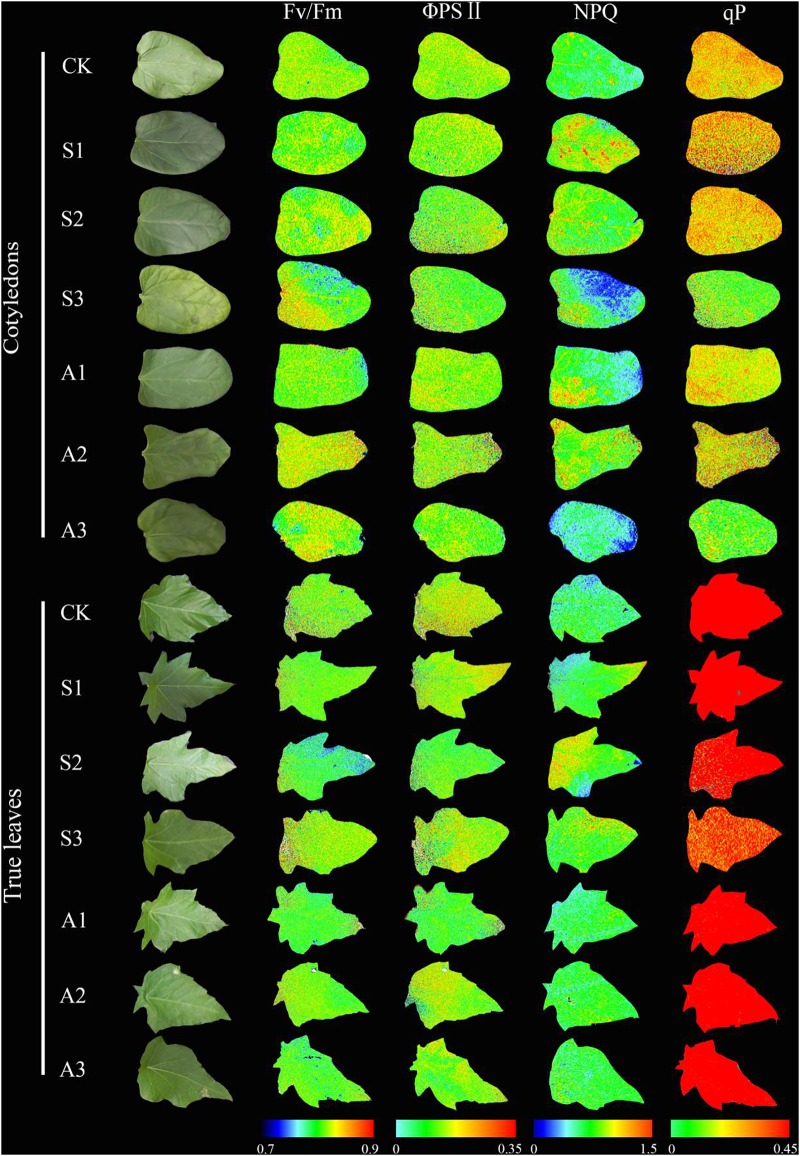
Representative fluocam false color images for Fv/Fm (maximum Photosystem II quantum yield), ΦPSII (effective Photosystem II quantum yield), NPQ (non-photochemical quenching), and q_p_ (Photochemical quenching coefficient) of *Ricinus communis* cotyledons and true leaves under salt-alkali stress.

With increasing salinity concentrations, NPQ values in cotyledons increased and then decreased, and the highest NPQ value (0.69) in the cotyledon was obtained at 80 mM salinity concentration. A similar trend was also found in alkalinity stress, but the NPQ value only presented a significant difference at 80 mM alkalinity stress (*P* < 0.05). NPQ value in true leaves significantly increased with increasing salinity (*P* < 0.05), and the highest value was found at 120 mM salinity, which was 26.4% higher than in the control group. NPQ in true leaves increased with the increasing alkalinity concentration, but did not present significant differences among the stress concentrations (*P* > 0.05, Figures 4G,H). NPQ values in true leaves were also much higher than those in cotyledons under both control and salt-alkaline stress. In addition, the differences in above four parameters (Fv/Fm, ΦPSII, q_p_ and NPQ) between cotyledons and true leaves under salinity-alkalinity stress were clearly shown by representative fluocam false color images (Figure 5).

### Correlation Analysis Between Biomass and Photosynthetic Indexes

Correlation analysis showed that the FW and DW of the cotyledons had a significant positive correlation with *P*_N_, *g*_s_, *T*r, Chl a, ΦPSII, and q_p_ under salt and alkali stresses. In terms of photosynthetic process, we found that the *P*_N_ of cotyledons had significant positive correlation with *g*_s_, *T*r, Chl a, and Car. In addition, a significant positive correlation was also shown in the ΦPSII and q_p_ under salt and alkali stresses (Table 2). Unlike cotyledons, the FW of true leaves had a significant positive correlation with *P*_N_, *g*_s_, *T*r, Car, and q_p_ under salt and alkali stresses. We also found that the *P*_N_ of true leaves had significant positive correlation with *g*_s_, *T*r, and Car. Moreover, a negative correlation between q_p_ and NPQ was found in true leaves under salt and alkali stresses (Table 3).

**Table 2:** Correlation analysis between biomass and photosynthetic indexes of cotyledons under salinity and alkalinity stresses.

Correlations	FW	DW	Water content	*P*_N_	*g*_s_	*T*r	Chl a	Chl b	Car	Fv/Fm	ΦPSII	q_p_	NPQ
FW	1												
DW	0.988^∗∗^	1											
Water content	0.918^∗∗^	0.904^∗∗^	1										
*P*_N_	0.931^∗∗^	0.925^∗∗^	0.714	1									
*g*_s_	0.937^∗∗^	0.960^∗∗^	0.782^∗^	0.941^∗∗^	1								
*T*r	0.953^∗∗^	0.940^∗∗^	0.783^∗^	0.971^∗∗^	0.949^∗∗^	1							
Chl a	0.914^∗∗^	0.958^∗∗^	0.801^∗^	0.890^∗∗^	0.980^∗∗^	0.886^∗∗^	1						
Chl b	0.482	0.566	0.443	0.421	0.549	0.34	0.64	1					
Car	0.581	0.655	0.309	0.759^∗^	0.786^∗^	0.649	0.804^∗^	0.589	1				
Fv/Fm	0.66	0.735	0.769^∗^	0.495	0.658	0.496	0.786^∗^	0.63	0.51	1			
ΦPSII	0.834^∗^	0.842^∗^	0.852^∗^	0.697	0.859^∗^	0.774^∗^	0.866^∗^	0.4	0.505	0.739	1		
q_p_	0.874^∗^	0.868^∗^	0.847^∗^	0.780^∗^	0.888^∗∗^	0.862^∗^	0.866^∗^	0.269	0.519	0.663	0.975^∗∗^	1	
NPQ	0.029	0.057	0.348	−0.225	−0.026	−0.11	0.085	−0.14	−0.246	0.556	0.404	0.335	1

*^∗^Represented significant correlation (P < 0.05), ^∗∗^represented extremely significant correlation (P < 0.01).*

**Table 3:** Correlation analysis between biomass and photosynthetic indexes of true leaves under salinity and alkalinity stresses.

Correlations	FW	DW	Water content	P_N_	g_s_	*T*r	Chl a	Chl b	Car	Fv/Fm	ΦPSII	q_p_	NPQ
FW	1												
DW	0.753	1											
Water content	0.980^∗∗^	0.805^∗^	1										
*P*_N_	0.971^∗∗^	0.703	0.981^∗∗^	1									
*g*_s_	0.967^∗∗^	0.619	0.958^∗∗^	0.984^∗∗^	1								
*T*r	0.906^∗∗^	0.561	0.903^∗∗^	0.951^∗∗^	0.971^∗∗^	1							
Chl a	0.502	0.389	0.43	0.507	0.43	0.46	1						
Chl b	−0.505	−0.012	−0.499	−0.517	−0.626	−0.568	0.311	1					
Car	0.949^∗∗^	0.662	0.964^∗∗^	0.991^∗∗^	0.987^∗∗^	0.980^∗∗^	0.459	−0.554	1				
Fv/Fm	−0.303	0.182	−0.338	−0.418	−0.496	−0.461	0.27	0.818^∗^	−0.458	1			
ΦPSII	0.383	0.381	0.248	0.155	0.192	0.058	0.223	−0.012	0.086	0.361	1		
q_p_	0.789^∗^	0.385	0.716	0.726	0.735	0.601	0.44	−0.56	0.663	−0.322	0.464	1	
NPQ	−0.705	−0.208	−0.673	−0.754	−0.752	−0.66	−0.458	0.578	−0.712	0.615	−0.057	−0.871^∗^	1

*^∗^Represented significant correlation (P < 0.05), ^∗∗^represented extremely significant correlation (P < 0.01).*

## Discussion

**FIGURE 6:**
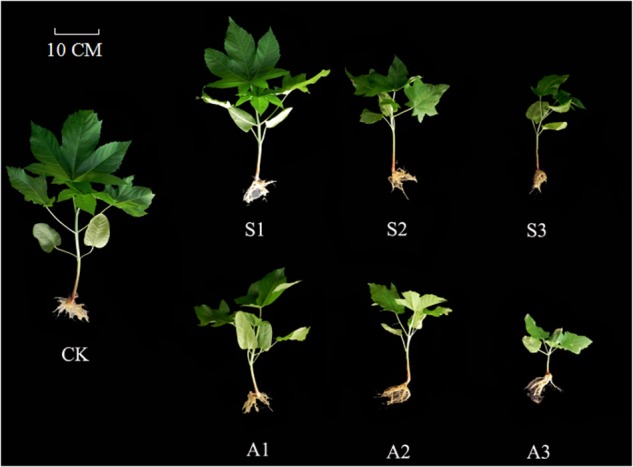
*Ricinus communis* seedlings under salt and alkali stresses. **CK**: control treatment. **S1**: 40 mM NaCl treatment. **S2**: 80 mM NaCl treatment. **S3**: 120 mM NaCl treatment. **A1**: 40 mM NaHCO_3_ treatment. **A2**: 80 mM NaHCO_3_ treatment. **A3**: 120 mM NaHCO_3_ treatment.

Generally speaking, early seedling stage is one of the most important and also sensitive stages during plant development in salt-alkali stress conditions (Abogadallah, 2010). Understanding the response of cotyledon and true leaf to such abiotic adversity stress is particularly crucial for clarifying the physiological adaptive strategies of *Ricinus communis* establishment during seedling stage. In the present study, weights of true leaves decreased more than those of cotyledons under salinity-alkalinity stress (Figure 6). These results indicate that the tolerance in castor cotyledons to salinity-alkalinity stress was much stronger than that in true leaves. Similar results were also found in Jatropha by Gao et al. (2013). Castor cotyledon, unlike most other plants, can be retained in the seedling and does not off for more than a month during early seedling stage. It can provide amounts of photoassimilate for plant growth (especially true leaves). The higher tolerance of cotyledon to salt-alkali stress may be an adaptive strategy of this species to cope with adverse environmental factors and guarantee the following of true leaf normal growth. Studies carried out by Wang et al. (2012) also indicated that cotyledons of *Vicia cracca* were more tolerant to NaCl stress than shoots. Moreover, much stronger inhibition in cotyledons and true leaves weights were detected under alkalinity stress, potentially due to the destructive effect of a high pH level, which directly led to ion imbalance and metabolic disorders (Yang et al., 2007; Lin et al., 2016).

Losing water is a rapid way for plants to adjust their osmotic balance in response to osmotic stress under salt-alkali stress conditions. In the present study, WC in cotyledons and true leaves were both affected by a higher salinity level. However, the WC is not significantly different from that in the control group at lower salinity concentration (40 mM), indicating that when the stress concentration was relatively low, the roots of castor bean can maintain a certain water adsorption ability to stabilize the intracellular environment (Chen et al., 2005; Lin et al., 2014). However, the WC in cotyledons and true leaves decreased much more markedly under alkalinity stress. The reason may be that a high pH level caused by alkalinity stress strongly inhibited water absorption by the roots, and the castor bean seedlings synthesized a higher amount of matter to resist stress so that more water was consumed. Similar results have also been reported in cotton plants by Chen et al. (2010).

Photosynthesis is one of the most important indicators of physiological sensitivity to abiotic stress (e.g., Higher or lower temperature, soil salinization, higher CO_2_ or O_3_) (Chaves et al., 2009). In this study, salinity-alkalinity stress significantly reduced the photosynthetic capacity in cotyledons and true leaves, and the decrease in the *P*_N_ value of cotyledons was much greater than that of true leaves. The reason for this phenomenon is that the cotyledons of castor bean can accumulate a large amount of Na^+^, and restrict Na^+^ transportation to the true leaves, which ultimately alleviates the damage effects to photosynthesis system of true leaves caused by toxic ions (Wang X. et al., 2015). In addition, the Chl a and Car contents in cotyledons decreased under salinity-alkalinity stress. These results indicated that the metabolic disorders caused by some salt ions (e.g., Na^+^) increased the activity of Chl-degrading enzymes, inhibited Chl synthesis, and disrupted the chloroplast structure (Shi and Zhao, 1997; Djanaguiraman and Ramadass, 2004). The negative effects ultimately led to a decrease in chlorophyll contents of cotyledon. These results were in agreement with a previously published study on the quinoa (Ruffino et al., 2010). Moreover, we also found that no photosynthetic pigments contents were changed with the increasing salinity-alkalinity concentration in true leaves. The main reason for this finding is that cotyledons can reduce some toxic ion contents, such as Na^+^ in true leaves (Na^+^ compartmentalization and maintain high K^+^ level) to alleviate photosynthetic pigments degradation and synthesis inhibition caused by the ion disorder (Zhou et al., 2014), which deserves further research. The Chl a, Chl b, and Car contents in true leaves were all much higher than those in cotyledons under both control and salt/alkali stress. This finding can also explain why the *P*_N_ value in true leaves was higher than that in cotyledons under such conditions.

The use of chlorophyll fluorescence as a rapid non-destructive method to examine photosynthetic performance and stress feature in plants is now widespread in many fields (Jiang et al., 2017). Fv/Fm can be used to estimate the maximum quantum yield of Q_*A*_ reduction and reflect the photoinhibition of PSII (Baker and Rosenqvist, 2004; Hichem et al., 2009). However, The slight decrease of Fv/Fm in castor bean seedlings under salt and alkali stresses is likely due to the reversible inactivation or downregulation of PSII [such as photosynthetic electron transport and RuBP carboxylase/oxygenase (Rubisco) activity] rather than the photodamage of PSII (Liu and Shi, 2010; Hanachi et al., 2014). ΦPSII was assessed from the quantum efficiency of photochemical energy dissipation which relates to the utilization of photons absorbed by the PSII antennae (Hanachi et al., 2014; Ding et al., 2016). Photochemical quenching (q_p_) indicates the oxidation reduction state of the primary acceptor (QA) for PSII and reflects the PSII susceptibility to photoinhibition (Lu and Lu, 2004; Chen et al., 2006). In our study, ΦPSII and q_p_ in cotyledons both decreased at the highest salinity and higher alkalinity concentrations, indicating an increase in the susceptibility to photoinhibition of cotyledon. These results also reflected that electron transport through PSII and the susceptibility to photoinhibition in cotyledons was more easily affected by alkalinity stress. The reason was that a high pH level caused a series of damages, such as destroying of photosynthetic machinery and primary electron acceptors, weakening PSII activity, and reducing the photochemical reaction (Liu and Shi, 2010). We also found that ΦPSII in true leaves did not change under salinity-alkalinity stress, compared to that in the control group, while the value was much lower in cotyledons, indicating that the PSII function in the true leaves was better than cotyledons under salinity-alkalinity stress. Although the PSII function in true leaves was not affected by increasing salinity-alkalinity, the susceptibility to photoinhibition increased with rising salinity-alkalinity, and it should be reflected in the downtrends in q_p_ values with the increasing stress concentration.

NPQ reflects the part of light energy absorbed by PSII antenna pigments, which cannot be used for photosynthetic electron transport and dissipate in the form of heat (Chen et al., 2011). In the present study, NPQ in cotyledons increased first and then decreased with increasing salinity-alkalinity. Previous studies have reported that an increase in the NPQ had been thought to be an energy dissipation mechanism that protected the photosynthetic apparatus against excess light under salinity-alkalinity stress (Demmig-Adams and Adams, 1992; Zribi et al., 2009). Therefore, our findings reflected the protective or regulatory mechanism in cotyledons to avoid the photodamage of the photosynthetic apparatus. However, the downtrends of NPQ in cotyledons at the highest salinity-alkalinity level was not because of the enhancement of photosynthetic electron transport, but due to the decrease in the heat dissipation ability caused by the damage to chloroplasts under salinity-alkalinity stress, such as thylakoid swelling, envelope decomposition, and instability of pigment-protein complexes (Lu et al., 2016). The specific mechanism needs further research. Compared with cotyledons, true leaves of castor bean had higher NPQ values under salinity-alkalinity stress. This result suggests that true leaves have a stronger heat dissipation ability to better protect the photosynthetic apparatus. In addition, NPQ in true leaves at the highest salinity stress was higher than that at alkalinity stress, indicating a lower heat dissipation ability under alkalinity stress. The result also indicated that PSII of true leaves suffered more damage under alkalinity stress than salinity stress because of the high pH level, which needs further research.

## Conclusion

This paper first reported the different adaptation strategies (from the viewpoint of growth, photosynthesis and chlorophyll fluorescence) of cotyledon and true leaf in *Ricinus communis* under salt stress and alkali stress. The results clearly showed that alkali stress caused more damage to castor bean seedlings than salt stress. The decrease in the biomass of cotyledons is lower than that in true leaves. Salt-alkali stress only reduced photosynthetic pigments and ΦPSII in cotyledons, but did not affect those in true leaves. Additionally, Fv/Fm and NPQ both decreased in cotyledons but increased in true leaves with increasing salinity-alkalinity. Different physiological responses and adaptive strategies were found in cotyledons and true leaves of this species under salt-alkali stress. The results will help us develop a better understanding of the adaptation mechanisms of cotyledon and true leaf during early seedling stage of castor bean plant, and provide new insights into the function of cotyledon in *Ricinus communis* under salt-alkali stress conditions.

## Author Contributions

YW wrote the manuscript. WJ did the part of the experiments. XH did the experiments. XP analyzed the data. XY revised the grammar. ZZ and JL revised the manuscript.

## Conflict of Interest Statement

The authors declare that the research was conducted in the absence of any commercial or financial relationships that could be construed as a potential conflict of interest.
